# Tecnologías asistivas para pacientes ancianos con demencia: perspectivas desde la bioética de los cuidados en salud

**DOI:** 10.18294/sc.2023.4488

**Published:** 2023-10-03

**Authors:** Isis Laynne de Oliveira Machado Cunha

**Affiliations:** 1 Doctora en Bioética. Posdoctoranda, Pontifícia Universidade Católica do Paraná, Brasil. isis_laynne@hotmail.com Pontifícia Universidade Católica do Paraná Pontifícia Universidade Católica do Paraná Brazil isis_laynne@hotmail.com

**Keywords:** Anciano, Demencia, Dispositivos de Autoayuda, Bioética, Empatía, Aged, Dementia, Self-Help Devices, Bioethics, Empathy

## Abstract

La demencia es actualmente una de las enfermedades más comunes que afecta a las personas mayores, siendo la séptima causa principal de muerte. Provoca pérdida de memoria, dificultad para razonar y, por consiguiente, dificultades para tomar y ejecutar decisiones, por lo que las tecnologías de asistencia y estimulación cognitiva son valiosos recursos en el proceso de cuidado. Desde una investigación teórica basada en la bioética de los cuidados en salud y en las investigaciones de Aline Albuquerque y Victor Montori, este artículo aborda, en primer lugar, el concepto de bioética en el cuidado de la salud, la atención centrada en el paciente y la idea de empatía clínica; en segundo lugar, se centra en el empleo de tecnologías asistivas para el cuidado de adultos mayores con demencia y, por último, plantea la discusión sobre si el proceso de atención podría ser considerado como una tecnología sanitaria.

## Abstract (en)

Dementia is currently one of the most common diseases affecting elderly people, ranking seventh among leading causes of death. As it causes memory loss, difficulties in reasoning, and resulting difficulties in making and executing decisions, assistive technologies and cognitive stimulation are valuable resources in the care process. Based on a theoretical inquiry into the bioethics of health care, and drawing on the work of Aline Albuquerque and Victor Montori, this article focuses on three main issues: first, the concept of bioethics in health care, patient-centered care, and the idea of clinical empathy. Second, it looks at the use of assistive technologies in caring for elderly patients with dementia. Lastly, we offer a discussion of whether the care process could be considered a health technology.

## INTRODUCCIÓN

El envejecimiento de la población ha despertado preocupación en relación con la aparición de enfermedades crónicas cada vez más limitantes. A pesar de no ser una consecuencia inevitable del proceso de envejecimiento, la demencia es actualmente una de las enfermedades más comunes que afecta a las personas mayores, siendo la séptima causa principal de muerte^1^.

En 2017, la Organización Mundial de la Salud (OMS) estimó que alrededor de 50 millones de personas en todo el mundo vivían con demencia, y se esperaba que ese número se triplicara para 2050^2^, debido a una serie de factores poblacionales y demográficos que contribuyen al aumento de dicho número. Esto genera una enorme preocupación con relación a los cuidados en salud y la preservación de los derechos de estas personas, y a la necesidad de mantener una vida digna^3^, especialmente en la vejez, ya que en esta etapa el cuidado del cuerpo y de la salud se hacen más necesarios y costosos^4^.

La demencia, cuando está en su etapa más avanzada, afecta las habilidades mentales para la toma de decisiones, lo que repercute en el ejercicio de la autonomía personal y el desarrollo de actividades cotidianas, requiriendo un alto nivel de cuidado^5^. Cabe destacar que uno de los predictores de la calidad de vida se refiere a la posibilidad de realizar personalmente las actividades del día a día, y al respeto por la autonomía en la toma de decisiones, especialmente en lo que se refiere al cuidado en salud^6^^,^^7^. En este sentido, los avances tecnológicos pueden contribuir a mantener la vida autónoma durante el mayor tiempo posible. 

Las definiciones de tecnología varían y el término se puede utilizar como sinónimo de herramienta o máquina, así como referirse a la aplicación del conocimiento organizado a tareas prácticas por sistemas ordenados por personas y máquinas^8^. La tecnología asistencial puede ser descrita como todo dispositivo técnico que ayuda y facilita la realización de actividades diarias, reduce el trabajo de las personas cuidadoras y proporciona mayor autonomía a las personas mayores. Ejemplos de tecnologías asistivas incluyen dispositivos GPS para facilitar la ubicación de personas con demencia, robots que contribuyen a la estimulación cognitiva y social, así como juegos que permiten a las personas con demencia participar en el ámbito virtual^9^. Sin embargo, el uso de estas tecnologías también plantea discusiones éticas sobre el impacto individual y social, para la adaptación de pacientes mayores que viven con demencia^8^.

Teniendo en cuenta que la demencia conlleva la pérdida de memoria, dificultad para razonar y, por consiguiente, dificultades para tomar y ejecutar decisiones, las tecnologías de estimulación cognitiva son valiosos recursos en el proceso de cuidado, ya que pueden aumentar la plasticidad neuronal, prolongar el mantenimiento de las funciones mentales, motoras y sociales, así como la capacidad de pensar con claridad y tomar decisiones^10^. 

A pesar del gran número de personas mayores que viven con demencia en Brasil^4^, existen pocos estudios que aborden el uso de tecnologías asistivas dirigidas a este grupo, lo que subraya la necesidad de desarrollar más investigaciones y discutir aspectos éticos relacionados con el uso de tales mecanismos^9^. 

La discusión es importante desde el punto de vista bioético, ya que se trata de un área multidisciplinaria que analiza éticamente aspectos de la vida humana en sus campos más amplios, desde perspectivas que involucren conceptos ambientales, biológicos, sanitarios, sociales y valores éticos adoptados en un determinado contexto^11^. El tema es aún más relevante en lo que respecta al campo de la bioética de los cuidados de salud, ya que se orienta por el cuidado centrado en el paciente, en su deseos y preferencias; reconoce la existencia de asimetrías de poder en la relación entre el profesional de la salud y el paciente; y se basa en los derechos humanos aplicados al contexto del cuidado de la salud, lo que conlleva prescripciones éticas y jurídicas para las personas implicadas en el cuidado^12^. 

El uso de tecnologías asistivas dirigidas a pacientes mayores con demencia puede contribuir a la promoción de la autonomía y una mejor calidad de vida o, en determinados casos, puede aumentar la vulnerabilidad, especialmente cuando se intenta reemplazar el cuidado humano por el uso de estas tecnologías^9^. 

Ante este escenario, desde una investigación teórica basada en la bioética de los cuidados en salud, este artículo propone analizar el uso adecuado de tecnologías asistivas en el campo de los cuidados de salud, con el objetivo de señalar un camino ético, jurídico y social apropiado para mejorar la calidad de vida y salud de pacientes mayores con demencia^12^^,^^13^^,^^14^^,^^15^, y cómo el uso adecuado de tecnologías puede contribuir a la promoción de la autonomía, al aumento de los cuidados de salud y a la mejora de la calidad de vida de este grupo.

## METODOLOGÍA

Este trabajo se sustenta en una investigación teórica, la cual se distingue de la revisión bibliográfica al ser estructurada sobre una teoría preexistente en un determinado campo de investigación. La esencia de este tipo de investigación es adoptar una línea teórica que permita sustentar el análisis y el debate sobre un tema concreto. El marco teórico no solo guía las direcciones de la investigación, sino que también proporciona los fundamentos para avalar su solidez. De esta manera, actúa como pilar sobre el que se erige el estudio^16^.

Para este artículo en particular, se adoptó como marco teórico la bioética centrada en el cuidado de la salud, tomando como referencia investigaciones llevadas a cabo por Aline Albuquerque, complementadas con los estudios de Victor Montori con relación a la atención enfocada en el paciente.

La elección de este marco teórico para el análisis del empleo de tecnologías asistivas en el ámbito de la salud de adultos mayores con demencia se fundamenta en las recientes contribuciones bioéticas orientadas al cuidado de la salud, que destacan que el proceso de atención debe responder a las necesidades concretas del paciente.

El desarrollo del artículo se divide en tres secciones. La primera, aborda el concepto de bioética en el cuidado de la salud, detallando su contribución teórica y sus fundamentos, para luego transitar por la atención centrada en el paciente y la idea de empatía clínica. La segunda sección se centra en el empleo de tecnologías asistivas para el cuidado de adultos mayores con demencia. Por último, la tercera sección plantea la discusión sobre si el proceso de atención podría ser considerado, también, una tecnología sanitaria.

## EL PARADIGMA DE LA BIOÉTICA DE LOS CUIDADOS EN SALUD

En primer lugar, es importante destacar que la llamada “bioética de los cuidados en salud” se refiere a una corriente de la bioética que, en contraposición a otras, enfatiza la relación humana entre el profesional de la salud y el paciente, valorando la relación entre ambos, así como la centralidad del paciente en el cuidado. Utiliza la experiencia del paciente, junto con el papel del profesional de la salud, como fuente de sus aportes teóricos, buscando el bienestar y la calidad de vida del paciente^12^. 

También hay que considerar que el término “paciente” se refiere a la persona que está bajo el cuidado de salud, que puede estar enferma o sana, pero que busca una mejor atención para su salud y calidad de vida^17^. En este sentido, la palabra paciente no debe ser asimilada a la condición de pasividad, sino a su doble condición frente al proceso terapéutico, es decir, su vulnerabilidad y su centralidad^17^.

La bioética de los cuidados en salud se basa en el enfoque del *cuidado centrado en el paciente*, en su participación y empoderamiento, así como en la empatía clínica y en la aplicación de los derechos humanos al contexto de los cuidados de salud. Pueden ser citados como ejes teóricos la comunicación empática, la relación de camaradería entre profesional y paciente y la centralidad y empoderamiento de este último^12^. 

El cuidado centrado en el paciente denota una ruptura con el paradigma actualmente predominante, en el que el profesional de la salud, permeado por una serie de factores estructurales y económicos, muchas veces desatendidos en su práctica profesional diaria, se aleja del contacto cercano con el paciente y lo comprende como objeto de cuidado, otorgando mayor atención a la enfermedad y al conocimiento profesional, en detrimento de los conocimientos y experiencias personales del paciente^15^^,^^17^. Propone un cambio de conducta y desafía las prácticas y comportamientos profundamente arraigados, en el sentido de implementar la camaradería en el trabajo entre el profesional de la salud y el paciente^14^. 

En este sentido, el cuidado centrado en el paciente puede ser comprendido como una mirada más atenta al proceso de cuidar^17^, que debe ser amable y enfocado en las necesidades específicas del paciente, no solo en el intento de sanar enfermedades o de cumplir protocolos médicos, sino de adentrarse en su biografía, a través de la escucha atenta y la valoración individual^15^. Propone que las decisiones inherentes al cuidado de la salud deben ser guiadas por las necesidades, la voluntad y las preferencias del paciente, promoviendo su autonomía a través de su compromiso y la oferta de apoyos necesarios para la toma de decisiones relevantes que impactarán en su salud y calidad de vida^18^. 

La empatía clínica constituye uno de los pilares fundamentales de la bioética orientada al cuidado de la salud. Esta capacidad inherente al ser humano se despliega en, al menos, dos dimensiones: una cognitiva en la que el profesional de la salud comprende la perspectiva del paciente, y otra emocional en la que el profesional sintoniza con las “emociones del paciente”^15^. En resumen, la empatía implica conectarse con los sentimientos de la otra persona sin asumirlos como propios, sino comprenderlos y compartirlos. Su aspecto cognitivo requiere el uso de la capacidad imaginativa para acercarse a la perspectiva del otro. La empatía clínica no lleva a la fatiga empática o al agotamiento del profesional de la salud, ya que reconoce las necesidades del otro al mismo tiempo que es consciente de su propia diferencia con relación a la otra persona^19^. Es decir, hay una separación del sentimiento del otro con relación a la propia emoción. 

La empatía clínica implica tres componentes claves: la comprensión, la comunicación y la acción. La comprensión se refiere a entender lo que la enfermedad significa para el paciente, que requiere un diagnóstico preciso, un pronóstico y un proceso de toma de decisiones compartido. La comunicación tiene como objetivo aliviar la ansiedad del paciente y responder sus dudas, y es necesaria para que el profesional de la salud pueda comprender la situación real del paciente y verificar si la información proporcionada fue comprendida por el paciente. La acción está dirigida a maximizar los beneficios terapéuticos^19^. 

La empatía clínica es un concepto relacional, en el sentido de que es una experiencia orientada a la conexión con el otro^20^ y una habilidad humana que puede ser aprendida, aumentada o disminuida, dependiendo del entorno y la estructura organizacional en la que el profesional de la salud se encuentre^21^. Tiene un papel relevante en la bioética de los cuidados de salud, ya que implica tanto la noción de cuidar como la de curar, y puede complementar la medicina basada en la evidencia. Además, la función moral de la empatía clínica es promover la autonomía del paciente, un elemento fundamental en el proceso de toma de decisiones de personas mayores que viven con demencia.

La aplicación de los derechos humanos en el contexto de los cuidados de salud tiene como objetivo proteger la dignidad humana inherente al paciente, especialmente debido a su condición de vulnerabilidad. Los derechos humanos de los pacientes se refieren a un conjunto de derechos que los pacientes tienen cuando están bajo cuidados de salud^17^. Estos derechos incluyen el derecho a la vida, a la privacidad, a no ser sometido a tortura o a tratos inhumanos o degradantes, a la información, a la salud y el derecho a no ser discriminado^22^. 

En el contexto de los pacientes mayores^23^, este grupo se encuentra en una situación de vulnerabilidad aumentada^24^, debido a los prejuicios relacionados con la edad avanzada y el imaginario social de que la persona pierde la autonomía para gestionar su vida a medida que envejece, incluyendo las decisiones sobre cuidados de salud^25^. 

Además, los derechos humanos extraídos de normativas internacionales se aplican al campo de los cuidados de la salud^12^^,^^26^. Los derechos humanos de los pacientes definen un concepto que se deriva de la dignidad humana inherente a cada persona y aplican de forma neutral los principios de los derechos humanos universales y legalmente reconocidos, protegiendo tanto a pacientes como a profesionales de la salud y admitiendo limitaciones que puedan justificarse por normas de derechos humanos^26^.

A partir de la tipología obligacional presente en las concepciones inherentes a los derechos humanos, se establecen las obligaciones del Estado de respetar, proteger y garantizar dichos derechos, que se dividen en facilitar y proveer dichos derechos^27^. El incumplimiento de alguna de estas obligaciones resulta en una violación de los derechos humanos^17^. Este entendimiento es aplicable tanto al Estado como a la sociedad y, por consiguiente, a los profesionales que trabajan en el contexto de los cuidados de salud^17^. La concepción de los derechos humanos de los pacientes ofrece un medio para evaluar cuestiones sistémicas de obligaciones y responsabilidades, siendo un instrumento importante para la bioética de los cuidados de salud^12^. 

En síntesis, la bioética de los cuidados en salud es un nuevo enfoque seriamente comprometido con el establecimiento de una relación de colaboración entre el profesional de la salud y el paciente, con el objetivo de empoderarlo para que desempeñe un papel activo en el proceso de toma de decisiones sobre sus cuidados en salud^12^. 

Este enfoque puede aplicarse a los cuidados en salud de pacientes mayores que viven con demencia, ya que a medida que la enfermedad progresa, mayores son las necesidades de cuidados más atentos, sin que tales acciones restrinjan la autonomía personal y dignidad de tales personas. Por lo tanto, el cuidado debe estar atento a reconocer que al comprometerse las habilidades relacionadas a la toma de decisiones no implica la pérdida de derechos, sino que la voluntad y las preferencias de la persona deben ser respetadas en los diversos ámbitos de su vida, incluyendo sus cuidados en salud^4^. La bioética de los cuidados en salud, en estos casos, puede entenderse más allá del entorno hospitalario, aplicándose también a la relación establecida entre el cuidador y la persona mayor, ya que con el aumento de la edad, otras enfermedades y limitaciones acompañan los casos de demencia, lo que requiere acciones continuas dirigidas a sus cuidados en salud.

Con la evolución tecnológica se pueden incorporar nuevos mecanismos en los cuidados de salud para facilitar la autonomía de los pacientes mayores que viven con demencia, ya sea para la toma de decisiones o para la realización de actividades diarias, reduciendo la carga de las personas responsables de su cuidado. Sin embargo, el uso de estas herramientas puede enfrentar desafíos económicos y sociales, y se necesitan discusiones que aborden aspectos bioéticos. Por lo tanto, la bioética de los cuidados de salud puede utilizarse como una herramienta tecnológica para mejorar o guiar el uso de otras tecnologías, prácticas e instrumentos que impliquen el cuidado de la salud de personas mayores con demencia.

### Tecnologías asistivas y el cuidado de personas mayores con demencia

En la vejez, el estímulo cognitivo es imprescindible para posibilitar nuevas experiencias, nuevos desafíos y aprendizajes, que aumentan la plasticidad neural, incrementan la transmisión de información en el cerebro que compone la esencia del ser humano y distingue su personalidad y forma de actuar^28^. La cognición es un proceso multidimensional que admite que el cerebro continúe siendo ejercitado incluso en la vejez, a través de actividades que entrenan la memoria, la atención y demás funciones cerebrales, y minimizan los efectos de la debilidad cognitiva causada por el proceso demencial. Los estímulos pueden ser realizados de manera individual o en grupo, de forma presencial o a través de tecnologías de comunicación^29^.

Las tecnologías se están desarrollando para apoyar la toma de decisiones de personas con demencia. Algunos ejemplos pueden ser:

1. Sistemas de recordatorio, utilizados para recordar a las personas con demencia sus compromisos, tareas y medicamentos.
2. Aplicaciones de teléfonos móviles y tabletas, que ayudan a las personas con demencia a mejorar la cognición y la memoria, así como a recordar compromisos, tareas y medicamentos.
3. Dispositivos de monitoreo, utilizados para seguir la salud y el bienestar de una persona con demencia; pueden incluir monitores de actividad, monitores de frecuencia cardíaca y otros dispositivos.
4. Sistemas de navegación, utilizados para ayudar a las personas con demencia a orientarse en nuevos lugares.
5. Sistemas de comunicación, utilizados para ayudar a las personas con demencia a comunicarse con cuidadores y otros miembros de la familia.
6. Tecnologías cognitivas, que son sistemas, dispositivos y aplicaciones diseñados para mejorar el rendimiento cognitivo humano, incluyendo funciones como atención, memoria, razonamiento, percepción y toma de decisiones; tecnologías que pueden ayudar a las personas mayores a mantener su independencia^30^.

Además, las tecnologías cognitivas pueden incluir juegos y aplicaciones de entrenamiento, tecnologías de asistencia e interfaces cerebro-máquina, que permiten la comunicación directa entre el cerebro y una computadora u otro dispositivo, y sirven para ayudar a personas con discapacidades motoras o de comunicación a interactuar con el mundo digital y sistemas de inteligencia artificial, que están diseñados para imitar las funciones cognitivas humanas, como el aprendizaje, la toma de decisiones y el reconocimiento de patrones. Se utilizan en una variedad de aplicaciones, desde asistentes virtuales hasta diagnóstico médico. En resumen, las tecnologías cognitivas tienen como objetivo mejorar la cognición humana y, por lo tanto, tienen el potencial de mejorar la calidad de vida de muchas personas^31^.

También, la inteligencia artificial puede ayudar en el diagnóstico temprano, identificando los primeros signos de demencia; en el monitoreo de pacientes, alertando a cuidadores o profesionales de la salud cuando ocurren cambios significativos en el comportamiento o la salud de los pacientes; en el apoyo a la toma de decisiones sobre el cuidado de pacientes con demencia; en el uso de terapias personalizadas adaptadas a las necesidades y preferencias individuales del paciente; en la asistencia a tareas cotidianas y el recordatorio de compromisos; y en el monitoreo remoto, siguiendo el progreso del paciente^32^. 

Algunos dispositivos electrónicos, como celulares y computadoras, combinados con información, comunicación y prácticas de cuidado, pueden ser importantes tecnologías de mantenimiento de la salud, estimulación cognitiva, administración de demencia y aislamiento social. Su uso puede permitir la participación de las personas mayores con demencia en grupos de integración, junto con sus familiares, cuidadores y profesionales de la salud, para compartir miedos, angustias, prácticas de cuidado e intercambiar experiencias. En este sentido, dichos dispositivos se convierten en fuentes de apoyo^33^. 

Sin embargo, a través de dispositivos electrónicos, la comunicación y la multiplicación de la presencia y las experiencias subjetivas no siempre son bien comprendidas por aquellos que experimentan el proceso demencial. Existe una dificultad para comprender el funcionamiento de las pantallas y cómo las personas pueden comunicarse sin estar físicamente presentes. Además, está la falta de contacto físico, así como la dificultad de concentración y enfoque del paciente anciano con demencia en lo que las demás personas están compartiendo a través de los dispositivos electrónicos^33^. Por esta razón, es necesario que el cuidador esté presente para ayudar a la persona en el uso de equipos electrónicos que permitan la interacción con otras personas, lo que no necesariamente tiene que ocurrir cuando la interacción es presencial^29^. 

En esta línea, el cuidador emerge como una figura clave para la implementación de tecnologías asistivas y cognitivas en adultos mayores que conviven con demencia. Desempeñará el rol de mediador en la utilización de ciertas tecnologías, promoviendo así la autonomía e independencia del adulto mayor. Es esencial que el cuidador esté atento a las necesidades particulares del paciente, proporcionando una atención centrada en él, honrando sus deseos y preferencias e incentivando el empleo de tecnologías que potencien la autonomía. Por ende, el cuidado ofrecido debe estar en consonancia con el marco de la bioética orientada al cuidado de la salud.

Las personas mayores que viven con demencia pueden enfrentar varias dificultades en el uso de tecnologías asistivas y cognitivas, como la dificultad de acceso a las tecnologías, la complejidad en su uso que puede causar que se frustren o desistan, la falta de adaptación, ya que cada persona tiene especificidades que a menudo no son abarcadas por dichas tecnologías, los costos elevados, la necesidad de capacitación para el uso de nuevos dispositivos que no existían en la juventud de estas personas, y las limitaciones de memoria para seguir utilizando dispositivos electrónicos^34^. 

El continuo cuidado de las personas mayores con demencia contribuye al cuidado humanizado, en el que se valora la calidad de vida del individuo. Las intervenciones de profesionales de la salud y cuidadores estimulan y mantienen las capacidades mentales, fortalecen las relaciones sociales, brindan mayor seguridad y aumentan la autonomía del paciente, estimulando su identidad, autoestima y desempeño cognitivo y funcional^10^.

Las tecnologías asistivas y cognitivas pueden mejorar la calidad de vida de los pacientes mayores que viven con demencia, ya que les ayudan a mantener su independencia durante el mayor tiempo posible. Sin embargo, su uso debe estar vinculado a una buena práctica de atención, con la formación de una red de apoyo para que el individuo se sienta acogido y valorado.

### El proceso de cuidado como una tecnología de la salud

La actividad humana es un acto productivo, ya que modifica algo y produce algo nuevo. Muchas actividades están organizadas de forma interdependiente. En el campo de la salud, el “trabajo vivo en acto”, es decir, el trabajo humano en el momento exacto en que se ejecuta, determina cómo se llevará a cabo el proceso y la producción del cuidado^35^. La forma de organización de esta actividad se considera una tecnología blanda que integra maletín del saber técnico estructurado, la utilización de instrumentos propios de la actividad y las relaciones entre las personas implicadas en el cuidado.

En la producción del cuidado, estos saberes se utilizan de diferentes maneras, según la relación establecida entre el profesional de la salud, el cuidador y el paciente^35^. De esta manera, dependiendo de los actores implicados, el proceso de cuidado puede ser una tecnología blanda centrada en las relaciones y en la comprensión de las necesidades del paciente o, por el contrario, centrada en el uso de instrumentos o en el saber técnico en detrimento del paciente.

El proceso de cuidado es complejo e integra factores clínicos, ambientales y sociales que se acumulan y se articulan, haciendo que el acto de cuidar vaya más allá de los aspectos inherentes solo a la enfermedad. Los pacientes existen en la intersección de circunstancias sociales, personales y clínicas y, por lo tanto, pueden enfrentar múltiples factores complicadores. En general, estos factores se analizan de manera aislada y a través de su forma (clínica, socioeconómica o cultural) en lugar de sus funciones en la complejidad del cuidado. Pocas investigaciones examinan de manera integral cómo las combinaciones de factores interactúan para impactar a los pacientes^36^.

Los individuos se adaptan o sucumben a la complejidad de factores que los rodean con el tiempo, lo que omite la acumulación de factores complicadores y la posibilidad de resiliencia del paciente. Como ejemplos, se pueden citar: la carga de trabajo del paciente, referida a las tareas y responsabilidades con las que las personas lidian en su día a día, que incluyen el trabajo, la familia, el autocuidado y otras prioridades^37^^,^^38^; o la capacidad del paciente, vinculada a las habilidades, recursos o disposición para manejar las demandas, que incluyen el funcionamiento físico y mental, los recursos socioeconómicos, el apoyo social, la alfabetización y las actitudes y creencias^39^. Por lo tanto, una capacidad excesiva o baja puede desafiar por sí sola a los pacientes, causando dificultades de acceso, falta de adherencia, baja calidad de vida, entre otros problemas. Un desequilibrio entre la carga de trabajo que excede la capacidad puede considerarse uno de los principales factores de interrupción en la atención, el autocuidado y los resultados^36^.

La adopción de un modelo tecnológico de cuidado debe considerar necesariamente aspectos externos a la enfermedad, con el fin de alcanzar la experiencia, los impactos de las actividades desarrolladas por el paciente, sus creencias y la red de apoyo, de modo que el proceso de cuidado tenga un impacto positivo en su salud y calidad de vida.

Las tecnologías leves pueden ser representadas por actos de acogida, formación de vínculo y búsqueda de la promoción de la autonomía de los pacientes, a través de un diálogo abierto y una escucha activa y calificada^40^. Además del conocimiento técnico, de los instrumentos de trabajo y de las tecnologías más estructuradas, es necesario prestar mayor atención al papel de la relación, como tecnología leve presente en el acto de cuidar^41^. Este componente es fundamental para comprender que el cuidado en salud siempre es relacional y que desvalorizar este aspecto trae perjuicios para todo el proceso terapéutico.

La realización del trabajo de cuidado en salud basado en tecnologías leves, relacionales y empáticas, de acuerdo con la bioética de los cuidados en salud, contribuye a la formación de una línea de cuidado que, si se integra a todo el espectro de servicios de salud, tiene el poder de satisfacer diversas necesidades de los pacientes^41^. 

Los factores mencionados muestran la urgente necesidad de abandonar el paradigma de cuidado centrado en la enfermedad, adoptando un cuidado centrado en el paciente, tanto en la relación entre profesionales de la salud y pacientes, como en los modelos organizativos de gestión y políticas públicas. Esto contribuirá al uso adecuado de tecnologías asistivas, para que estas sirvan a las necesidades reales de cada paciente anciano que vive con demencia.

El uso de tecnologías de asistencia difícilmente alcanzará su objetivo final si no se conecta con el paradigma de la bioética de los cuidados en salud. Así, este paradigma se convierte en un importante instrumento y referente para la implementación de tecnologías asistivas y tecnologías ligeras en el cuidado de personas mayores que viven con demencia, ya que enfatiza la necesidad de respetar los derechos, aplicar la empatía clínica y establecer una relación respetuosa que apunte al cuidado centrado en el paciente.

## CONCLUSIONES

El mundo está en constante cambio, exigiendo el desarrollo de nuevas tecnologías y formas de relación. En los cuidados en salud, las tecnologías han sido importantes herramientas para mejorar la calidad de vida. Sin embargo, el uso de dichas herramientas y acciones realizadas por los cuidadores, precisan adaptarse a las necesidades sanitarias, económicas, sociales y también individuales de cada paciente.

El cuidado de pacientes mayores que viven con demencia ha planteado desafíos para familiares, cuidadores, profesionales de la salud y para la sociedad en general. Las tecnologías asistenciales y cognitivas han tenido un papel relevante para mantener la autonomía y mejorar la calidad de vida del paciente y demás actores implicados en su cuidado. Sin embargo, no pueden desvincularse de la empatía clínica, de la atención centrada en el paciente, del respeto a sus derechos humanos y la promoción de la autonomía.

En este sentido, los aportes traídos por la bioética de los cuidados en salud pueden ser considerados como tecnologías de cuidado, relevantes y necesarias que, asociados a tecnologías asistenciales y cognitivas, representan un camino ético, jurídico y social adecuado para mejorar la calidad de vida y salud de pacientes mayores con demencia.
